# CAR-T cell therapy for hematological malignancies: Limitations and optimization strategies

**DOI:** 10.3389/fimmu.2022.1019115

**Published:** 2022-09-28

**Authors:** Jiawen Huang, Xiaobing Huang, Juan Huang

**Affiliations:** Department of Hematology, Sichuan Academy of Medical Sciences and Sichuan Provincial People’s Hospital, University of Electronic Science and Technology of China, Chengdu, China

**Keywords:** CAR-T cell therapy, hematological malignancies, immune evasion, tumor microenvironment, toxicity

## Abstract

In the past decade, the emergence of chimeric antigen receptor (CAR) T-cell therapy has led to a cellular immunotherapy revolution against various cancers. Although CAR-T cell therapies have demonstrated remarkable efficacy for patients with certain B cell driven hematological malignancies, further studies are required to broaden the use of CAR-T cell therapy against other hematological malignancies. Moreover, treatment failure still occurs for a significant proportion of patients. CAR antigen loss on cancer cells is one of the most common reasons for cancer relapse. Additionally, immune evasion can arise due to the hostile immunosuppressive tumor microenvironment and the impaired CAR-T cells *in vivo* persistence. Other than direct antitumor activity, the adverse effects associated with CAR-T cell therapy are another major concern during treatment. As a newly emerged treatment approach, numerous novel preclinical studies have proposed different strategies to enhance the efficacy and attenuate CAR-T cell associated toxicity in recent years. The major obstacles that impede promising outcomes for patients with hematological malignancies during CAR-T cell therapy have been reviewed herein, along with recent advancements being made to surmount them.

## 1 Introduction

In recent years, cellular immunotherapy has emerged as one of the leading areas of on-going researches and clinical therapies. Allogeneic hematopoietic stem cell transplantation (HSCT) is one of the oldest forms of cellular immunotherapy, and it forms the theoretical basis for cellular immunotherapy and provides us the ability to harness immune system to eradicate malignancies. The underlying curative mechanism associated with HSCT against hematological malignancies is the graft-versus-leukemia (GVL) or graft-versus-tumor (GVT) effect (i.e., donor immune cells against abnormal leukemia/tumor cells in patient’s body) (1, 2). The ability of immune cells to recognize target cells to exert their function forms the basis of GVL or GVT. Therefore, a new era in cellular immunotherapy was initiated with the advent of genetically engineering T cell receptor to enable the targeting of a specific tumor antigen, which became the rational basis for chimeric antigen receptor (CAR) T-cell therapy (3, 4).

Since its first Food and Drug Administration (FDA) approval in 2017, CAR-T cell therapy has shown promising efficacy against certain hematological malignancies and even provides a curative treatment strategy for patients with advanced diseases, such as B cell acute lymphoblastic leukemia (B-ALL) or lymphoma. Currently, the major approved CAR-T cell therapy targets are B cell maturation antigen (BCMA) for multiple myeloma (MM) (5) and CD19 for various lymphoid malignancies including B-ALL and diffuse large B-cell lymphoma (DLBCL) (6–9).

Despite great success, barriers still remain that prevent the extension of CAR-T cell therapy beyond B cell/plasma cell driven hematological malignancies. Moreover, even for B cell driven cancers, the efficacy of CAR-T cell therapy is far from being satisfactory and great efforts should be done to improve clinical outcomes. As a newly emerged therapeutic strategy, newly developed direct modifications or combination therapies for CAR-T cell therapy are booming in recent years and they have deeply reshaped CAR-T cell therapy in hematological malignancies. These approaches can be categorized into four major aspects, aiming to address the four major hurdles for CAR-T cell therapy in hematological malignancies: tumor heterogeneity/antigen loss, immunosuppressive tumor microenvironment, CAR-T cell exhaustion (or poor persistence) and severe adverse-effects. This review will focus on recent novel approaches that have provided refinements and potential solutions to these limitations.

## 2 Target exploration and strategies to overcome antigen loss after CAR-T cell therapy

Despite the robust efficacy of the currently approved CAR-T cell products, follow-up studies still demonstrated a high rate of posttherapy relapse (10). The most common cause of resistance is *via* antigen loss/downregulation, which is for the selective pressure of CAR-T cells (11, 12). Such target antigen-negative relapses after CAR-T cell therapy usually arise from pre-existing antigen-negative cancer cells, or cells with altered/mutated antigen expression (12). Therefore, expanding the potential CAR-T cell target repertoires or overcoming antigen loss are key to ensure outcomes of CAR-T cell therapy. Besides the commonly adopted multi-specific CAR or dual CAR strategies, here we will discuss several novel aspects of antigen selections and methods to overcome antigen loss.

### 2.1 TCR-like CAR-T cells for intracellular antigens

Currently, CAR-T cell targets have mostly been restricted to extracellular tumor-associated antigens (TAA), which greatly limits the wider application of this promising therapy. This is a more pressing concern for hematological malignancies, as their mutation burden is lower, leading to a lower abundance of usable TAAs (13). Whereas, a rich range of intracellular oncoproteins have not been explored as targets. Therefore, T-cell receptor (TCR)-like CARs have been proposed as a means to utilize these targets. For example, intracellular protein Wilms tumor 1 (WT1) is an oncogene that promotes cell proliferation and differentiation. It is overexpressed in leukemia and lymphoma with limited expression in normal tissues (14, 15). The use of TCR-like WT1-CAR-T cell therapy has led to enhanced survival of leukemia-bearing mice (16, 17). Its principle is to combine an extracellular single chain variable fragment (scFv) that recognize the WT1 peptide-human leukocyte antigens (HLA) complex (as a full TCR do) with other CAR elements to generate TCR-like CAR-T cells (16, 17). Besides WT1, other intracellular antigen peptide-HLA complexes for CAR-T cell therapy have been investigated, including NY-ESO-1/HLA-A2 for potentially MM (18), HA-1H (derived from minor histocompatibility antigen 1, HMHA1)/HLA-A2 for chronic myelogenous leukemia (19), and SSX2/HLA∗0201 for acute myeloid leukemia (AML) (20). However, one of the difficulties of TCR-like CAR is that the target peptide and its associated HLA allele vary in different patients and cancers (e.g., WT1_235-243_/HLA-A*2402 (16) or WT1_126-134_/HLA-A*02:01 (17)). Hence, the applicability of TCR-like CAR-T cell needs to be evaluated on a case-by-case basis.

### 2.2 Monospecific CAR-T product with multiple targets

The increasing potential target repertoires enable the development of more comprehensive strategies against tumor heterogeneity and antigen loss. However, we should not limit our sights in simply identifying an excessive number of antigens and generating numerous CAR-T cell products to combat antigen loss. Previous studies suggest that the combination of multiple types of CAR-T cells may lead to growth competition, which severely impairs CAR-T cell proliferation (21). With proper design, a monospecific CAR-T product can be directed to multiple targets. For instance, the B-cell activating factor (BAFF) ligand binds to three different receptors BAFF receptor, BCMA, as well as transmembrane activator and CAML interactor (TACI) on mature B cells (22). Almost all B cell driven cancers express at least one of these receptors (23–27). Therefore, a single ligand-based BAFF-CAR-T cell product is capable of binding to all three of these receptors, which avoids the possibility of growth competition, enables broad antitumor activity and greatly minimizes the possibility of antigen escape (28).

### 2.3 Methods to prevent antigen loss

#### 2.3.1 Antigen independent targeting of cancer cells

Natural killer (NK) cells are ideal candidate for the elimination of antigen-negative cancer cells. Therefore, CAR-T cells can be converted into NK-like cells by pharmacologic agents to obtain the ability to overcome antigen loss and tumor heterogeneity. Bryostatin is an oxygenated macrolide that increases CD22 expression in ALL and chronic lymphocytic leukemia cells (29, 30). Combination therapy of bryostatin and CD22-CAR-T cells increases the CAR-T cell activity by increasing CD22 expression and sensitizing leukemia cells to CAR-T cell antigen-non-specific killing (31). However, this effect is limited to B-ALL cells, and does not affect Burkitt lymphoma cells.

#### 2.3.2 Preventing antigen loss

Perhaps the simplest way to prevent an antigen-negative relapse is to actively avoid antigen loss. Moreover, CAR-T cell activity is closely related to the expression level of its antigen on the cell surface. Therefore, treatments that maintain high antigen concentration on the cell surface may be able to prevent loss-of-antigen relapse. Combination of bryostatin and CD22-CAR-T cells discussed in previous section is an example of this strategy (31). In addition, gamma (γ)-secretase cleaves BCMA on cell surface (32) and therefore leads to loss of targets for BCMA-CAR-T cells. Inhibition of γ-secretase in murine models can reduce BCMA antigen loss and improve BCMA-CAR-T cell anti-tumor effect (33). Clinical trials combining γ-secretase inhibitors and BCMA-CAR-T cells are currently ongoing (NCT03502577). Similarly, all-trans retinoic acid (ATRA) can enhance BCMA expression on MM cells. It acts synergistically with the γ-secretase inhibitor to boost the function of BCMA-CAR-T cells (34).

Another direct way to prevent loss-of-antigen relapse is to artificially display antigen on cancer cells. Su et al. (35), designed a novel CAR-T cell engager which binds CD20 and displays CD19 on CD20^+^ lymphoma cells. In mice transplanted with JeKo mantle cell lymphoma cells, co-administration of CAR-T cell engager and CD19-CAR-T cells successfully eliminates both CD19^+^ and CD19^-^ lymphoma cells and prevent antigen-negative relapse. By changing the specificity of the engager, single type of CAR-T cells can be directed to different cancer subclones to overcome tumor heterogeneity. Moreover, by using single type of CAR-T cells, growth competition of multiple types of CAR-T cells can be avoided, further enhancing the anti-tumor activity.

## 3 Approaches to mitigate the immunosuppressive tumor microenvironment

**Figure 1:**
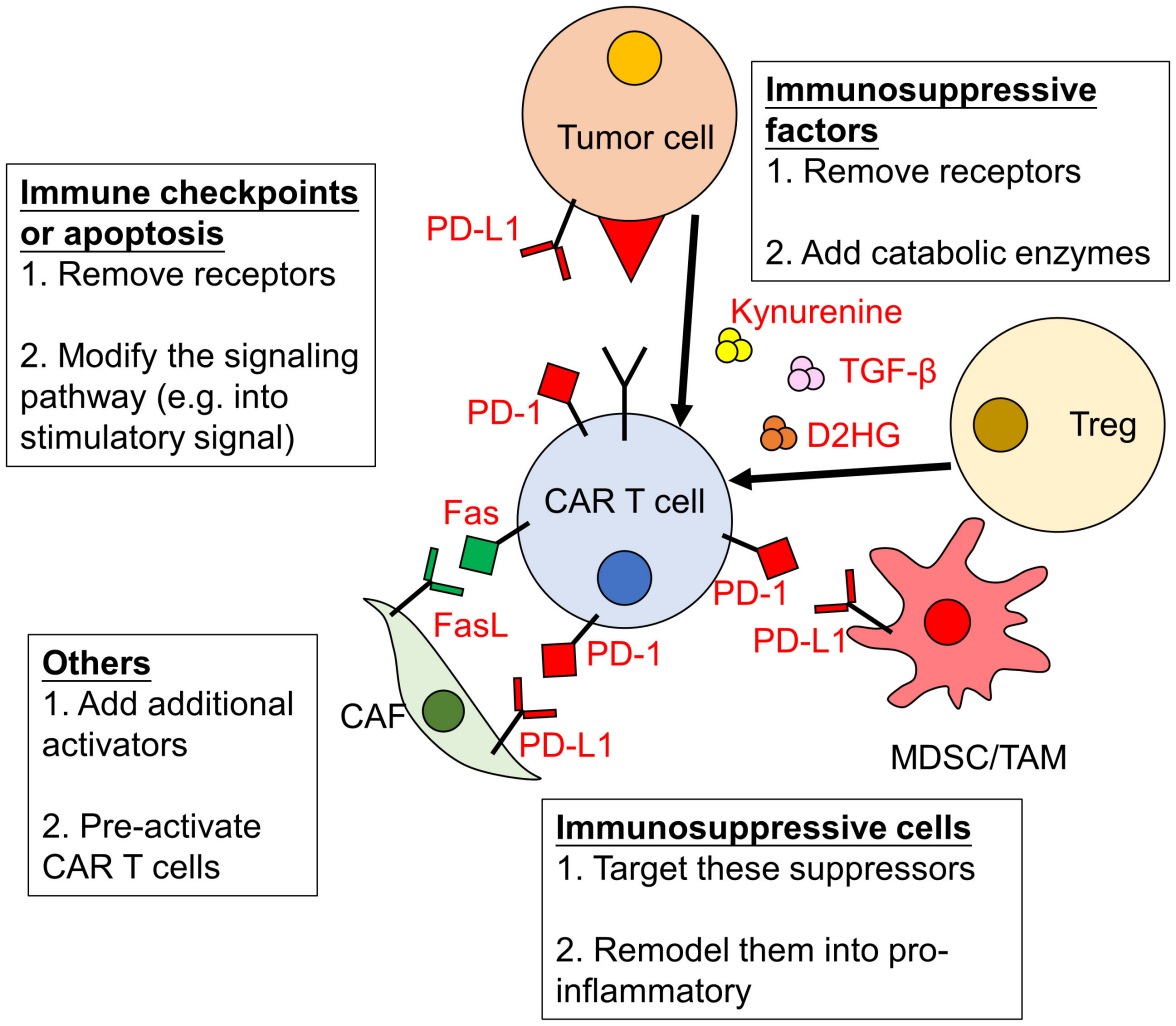
Immunosuppressive effects of the TME on CAR-T cells and strategies to overcome them. The activity of CAR-T cells can be severely impaired by the tumor microenvironment (TME). The immunosuppressive effects of the TME are due to increased level of immune checkpoints/inhibitors and immunosuppressive factors/cells. Strategies to overcome them include: (1) removing or modifying related signaling pathways; (2) eliminating or remodeling immunosuppressive cells and (3) enhancing the activation of CAR-T cells. PD-1, Programmed death-1; PD-L1, Programmed death-1 ligand-1; CAF, cancer-associated fibroblasts; MDSC, myeloid-derived suppressor cells; TAM, tumor associated macrophages; Treg, regulatory T cells; TGF-β, transforming growth factor -β; D2HG, D-2-hydroxyglutarate.

The tumor microenvironment (TME) can greatly impact the efficacy of CAR-T cell therapies. Numerous signals from cancer cells and surrounding cells have significant impacts on anti-tumor immunity (36–39). As the TME of hematological malignancy is mainly immunosuppressive, therefore it becomes another major cause of treatment failure. Indeed, the inhibitory immune checkpoint ligands Programmed death-1 ligand-1/2 (PD-L1/L2) are expressed in B cell driven malignancies (40). They bind to Programmed death-1 (PD-1) expressed on activated CAR-T cells, inhibiting their function. Recent study also showed that the TME of large B cell lymphoma is the key determinant of CD19-CAR-T cell activity (41). Several strategies have been proposed to overcome the immunosuppressive effects of the TME, including the blockade of immune checkpoints, promoting CAR-T cell activity and remodeling the TME (**Figure 1**).

### 3.1 Circumventing immune checkpoints during CAR-T cell therapy

By directly removing or blocking the immune checkpoints on CAR-T cells, their inhibitory effects might be avoided and therefore the anti-tumor effects of CAR-T cells are promoted. For example, the production of PD-1 resistant CD19-, CD133- and C-type lectin-like molecule-1 (CLL-1)-CAR-T cells achieved by the knockout of PD-1/PD-1 homolog genes can enhance CAR-T cell therapy (42–44). Recently, two relapsed/refractory AML patients who received HSCT and CD38-CAR-T cell therapy before showed continuous remission after receiving PD-1-silenced CLL-1-CAR-T cell therapy (45). Resistance to PD-1 signaling could also be acquired by CAR-T cells secreting soluble PD-1-blocking scFv (46). This strategy demonstrated matching efficacies against hematological malignancy models when compared to a combination therapy that incorporated CAR-T cells and an anti-PD1 antibody.

Besides direct blockade of PD-1, high levels of PD-L1 in the TME can also be leveraged by generating CD19-directed CAR-T cells that secrete soluble PD-1. These cells exhibited enhanced eradication efficiency against CD19^+^PD-L1^+^ cancer cells. It is thought that the increased efficacy associated with PD-1 secretion is due to a protective effect from apoptosis that enhances CAR-T cell persistence (47). Similar approaches are used for another immune checkpoint T-cell immunoglobulin and mucin-domain containing-3 (TIM-3) by generating TIM-3-CD28 fusion proteins in CD19- or CD138-CAR-T cells. It converts the immunosuppressive TIM-3 signaling to CAR-T cells immunostimulation (48, 49).

Other than PD-1 and TIM-3, Carnevale et al. (50), identified a novel immune checkpoint, RAS P21 Protein Activator 2 (RASA2), in T cells. RASA2 is a RAS GTPase-activating protein and is upregulated upon chronic stimulation. As demonstrated in multiple murine models including leukemia, CD19-CAR-T cells with RASA2-ablation experience increased RAS/MAPK signaling and activation, leading to enhanced and persistent anti-tumor activity (50).

However, it is notable that the effect of PD-1 removal in CAR-T cells should be carefully evaluated. Studies showed that using protein trap to remove PD-1 in CD19-CAR-T cells is associated with increased CAR-T cell activation, but also with diminished T cell survival and therefore reduced T cell cytotoxicity (51). Furthermore, these CAR-T cells also mature earlier and are quickly exhausted by T cell immunoreceptor with Ig and ITIM domains (TIGIT) upregulation (51).

### 3.2 Other approaches to avoid an immunosuppressive TME

#### 3.2.1 Targeting immunosuppressive effects (other than immune checkpoints)

Other than immune checkpoints like PD-1 signaling, numerous pathways have been linked to the modulation of CAR-T cell activity within an immunosuppressive TME. Although transforming growth factor beta (TGF-β) is a tumor suppressor in the premalignant stage, it promotes tumor transformation and growth at later disease stages (52). TGF-β also suppresses T cell function and therefore T cells with a dominant-negative form of the TGF-β receptor are resistant to TGF-β-mediated inhibition (53). As TGF-β is commonly expressed in NY-ESO-1^+^ cancers (53), the combination of TCR-like CAR-T cells targeting NY-ESO-1/HLA (18) with TGF-β blockade could be applicable for NY-ESO-1^+^ hematological malignancies as well.

Additionally, expression of Fas ligand in the TME can induce T cell apoptosis (37). Therefore, the expression of a dominant negative form of Fas receptor protects T cells in adoptive cell therapy (ACT) from Fas-induced apoptosis (54). Furthermore, Fas signal can be converted into T cell stimulatory signal *via* the transduction of T cells with Fas-4-1BB, which linked the Fas intracellular tail with costimulatory 4-1BB (CD137, Tumor necrosis factor receptor superfamily 9). This construct leads to increased T cell survival and enhanced T cell therapy against murine leukemia models (55). Similar strategies could be applied to CAR-T cells as well.

The TME modulates CAR-T cell metabolism as well. In the TME, kynurenine is often present, which inhibits glucose uptake and T cell function (56). Therefore, overexpression of kynureninase in CD19-CAR-T cells can protect them from immunosuppression by catabolizing kynurenine, and they exhibit excellent anti-tumor activity in mice bearing ALL cells (56). Similarly, the production of D-2-hydroxyglutarate (D2HG) by IDH1/2-mutated cancer cells can have critical implications for tumorigenesis and immunosuppression in the TME (38, 39). The overexpression of D2HG dehydrogenase (D2HGDH) in CD19-CAR-T cells decreases serum D2HG in mice bearing NALM6 leukemia cells with mutation IDH1 leading to enhanced T cell function and persistence (57).

#### 3.2.2 Pre-activating CAR-T cells prior to immunosuppressive TME exposure

The site of injection is also important for CAR-T cells activity. The intracerebroventricular (ICV) injection, rather than intravenous (IV) injection, of CD19-CAR-T cells is most effective (in terms of complete and durable eradication) for the treatment of murine model of central nervous system and systemic lymphoma (58). The superior CAR-T cell activity in this case is due to cerebrospinal fluid exposure of CAR-T cells in the ICV environment, which may alter cellular metabolism (58). Hence, the infusion of CAR-T cells to an immunostimulatory environment can pre-activate CAR-T cells and prevent immunosuppression.

### 3.3 Activating CAR-T cells to enhance their function in immunosuppressive TMEs

Rather than targeting immunosuppressive signaling, the activation of CAR-T cells can increase cytotoxicity within the TME. CD58 is a ligand for T cell costimulatory receptor CD2 (59, 60), which is frequently mutated in B cell driven malignancies. Notably, these mutations are associated with poor CD19-CAR-T cell therapy outcomes (61). The linkage of a second CAR construct (the CD2-signaling domain with a CAR intracellular domain) allows CAR-T cells to be activated in the absence of CD58 expression and increases their function (61). However, further investigations are needed to examine whether this results in T cell exhaustion.

In addition to CD58, other methods to promote CAR-T cell activity focus on improvements to their persistence, which are discussed in a separate section (“Approaches to prolong the persistence of CAR-T cells”).

### 3.4 Targeting and remodeling TME during CAR-T cell-therapy

Besides promoting CAR-T cell resistance to immunosuppression, directly targeting or even remodeling the TME provides an alternative approach to overcome its immunosuppressive effect.

#### 3.4.1 Targeting immunosuppressive TME

In MM, cancer-associated fibroblasts (CAFs) in the immunosuppressive TME play important roles in promoting tumor growth and inhibiting CAR-T cell function (62, 63). Therefore, Sakemura et al. (63), developed a dual-targeting CAR-T cell strategy, which targets both bone marrow CAFs (via SLAMF7 or Fibroblast activation protein/FAP) and MM cells (via BCMA). Under this condition, the anti-tumor activity of CAR-T cells is significantly improved. Besides CAFs, tumor associated macrophages (TAMs) and myeloid-derived suppressor cells (MDSCs) are also major contributors to the immunosuppressive effects in the TME *via* producing immunosuppressive cytokines and expressing immune checkpoints, which lead to T cell inactivation (64). Ultimately, these factors can cause CAR-T cell treatment failure. For example, it has been demonstrated that increased TAM infiltration is associated with a poorer CD19-CAR-T cell therapy outcome against B-cell non-Hodgkin’s lymphoma (65). Therefore, targeting MDSCs/TAMs by Gemtuzumab ozogamicin (66, 67), or Phosphoinositide 3-kinase (PI3K) blockade (68) are able to boost CAR-T cell activity against multiple cancer types, such as non-Hodgkin’s lymphoma and AML.

#### 3.4.2 Remodeling the immunosuppressive TME

TME remodulation is an alternative strategy for the prevention of immunosuppressive effects on CAR-T cells, which can even further increase CAR-T cell functions and contribute to sustained cancer remission. The use of CAR-T cells expressing immunostimulants or cytokines has been proposed for this purpose. IL-12 can enhance T cell activity, interferon γ (IFNγ) production and Th1 polarization, as well as serve as chemoattractant for various immune cells (69). Thus, CD19-CAR-T cells with IL-12-secreting ability could reshape the TME and induce robust T cell activity against lymphoma (70). However, constitutive IL-12 secretion is associated with toxic adverse effects, which necessitates the development of an inducible IL-12 secretion system (71). Recently, IL-12 nanostimulant-engineered CAR-T cells (INS-CAR T) provides a potential solution for this problem. In the presence of tumor antigen, IL-12-loaded human serum albumin (HSA) nanoparticles are released from INS-CAR T, allowing for local and controlled IL-12 release (and recruitment of other CAR-T cells) (72). Besides IL-12, IL-18 also enhances Th1 function, and IL-18-expressing CD19-CAR-T cells exhibit augmented activity in mice bearing ALL tumor (73).

Furthermore, whilst TAMs and MDSCs are known to be immunosuppressor, they have the plasticity to be transformed into M1-like pro-inflammatory phenotypes (74), which can enhance CAR-T cell activity. This transformation can be achieved by the co-administration of folate-targeted Toll-like receptor 7 agonist (FA-TLR7-1A) (75) or attenuated bacterial strain (*Brucella melitensis*) with TME homing ability (76). Engineered CAR-T cells secreting immunostimulatory RNA RN7SL1 are also able to inhibit MDSC development and activate immune cells in the TME (77). Although these approaches are initially designed to improve CAR-T cell activity against solid tumors, CD19-CAR-T cells are still used in these studies (75, 77). Therefore, it is worthy to investigate if similar strategies can be applied to certain hematological malignancies like lymphomas.

Recently, an immunostimulatory gene therapy using oncolytic viruses has been developed. Oncolytic viruses can specifically infect and kill tumor cells, promote TME remodeling and induce an enhanced immune response (78, 79). In murine models of human B-cell lymphoma, oncolytic adenovirus LOAd703-infected lymphoma cells exhibited enhanced immunogenic profiles with upregulated co-stimulatory molecules and chemokines, leading to enhanced CD19-CAR-T cell recruitment and anti-tumor activity (80). However, type I IFN expressed upon oncolytic virus infection may dampen the efficacy of CAR-T cells (81). Therefore, further investigations are required to optimize the combination therapy of oncolytic viruses and CAR-T cells.

## 4 Approaches to prolong the persistence of CAR-T cells

**Figure 2:**
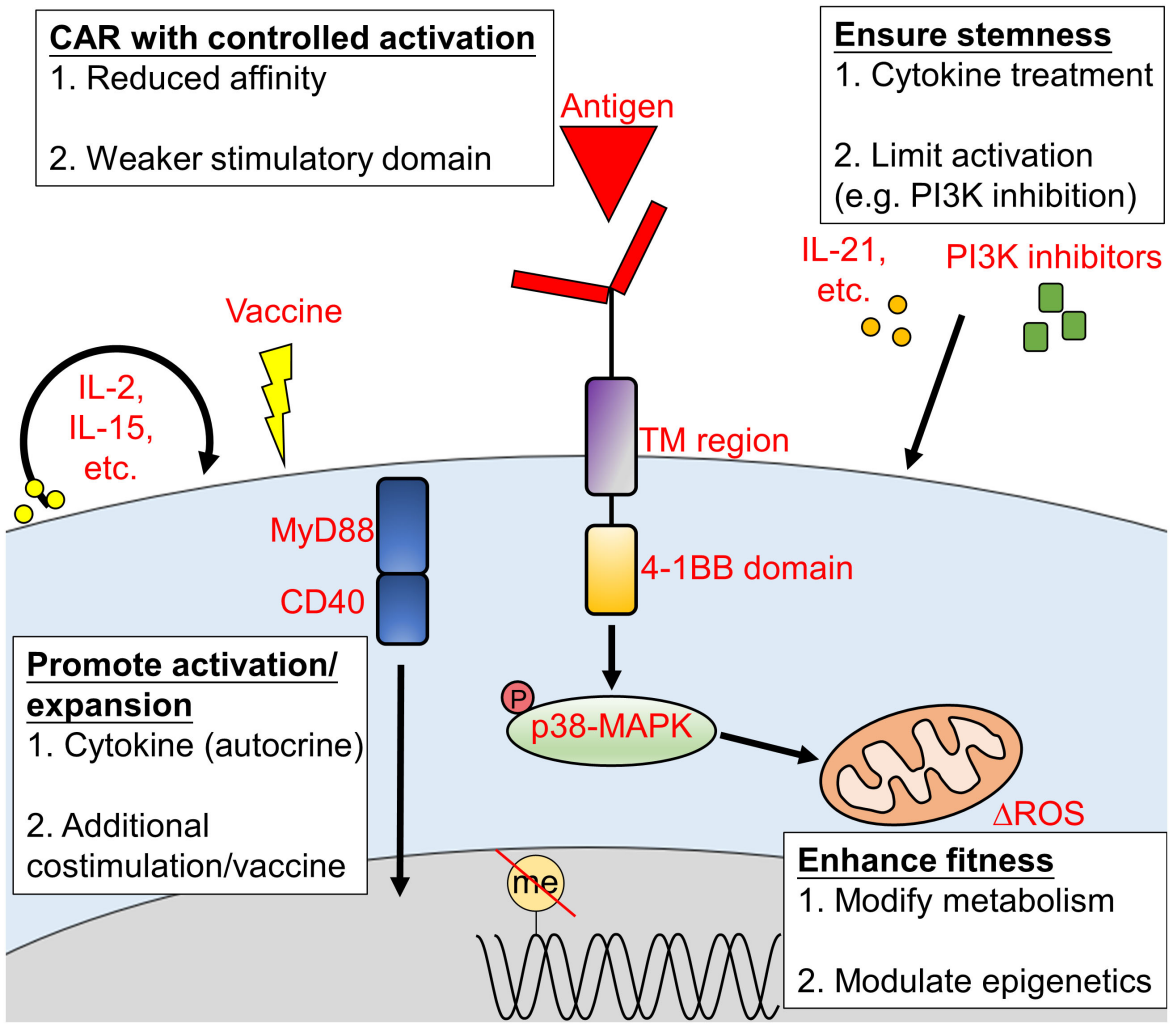
Approaches to promote CAR-T cell persistence *in vivo.* The persistence of CAR-T cells during treatment is crucial to achieve a durable and long-lasting outcome. CAR-T cell persistence can be extended *via* (1) the promotion of CAR-T cell activation or expansion; (2) enhanced CAR-T cell fitness; (3) proper CAR construct design and (4) increased CAR-T cells stemness. TM, transmembrane; ROS, reactive oxygen species.

The poor persistence of CAR-T cells is another major obstacle that stands in the way of long-term cancer remission of CAR-T cell therapy (82). This is often due to a combination of inter-related factors, including T cell exhaustion, transcriptional changes, metabolic changes and influences from the TME (83, 84). The persistence of CAR-T cells can be prolonged by enhancing CAR-T cell activity and expansion, improving fitness, reducing exhaustion, engineering the cells to have a less-differentiated state, altering their metabolism, and improving CAR design (**Figure 2**). These factors are inter-related. For example, cytokines that regulate T cell activity will alter the state of T cell differentiation and metabolism (85). The following sub-sections mostly focus on the major direct effects associated with each approach.

### 4.1 Ensuring CAR-T cell persistence by promoting their activity and expansion

#### 4.1.1 Cytokine-secreting/responsive CAR-T cells

Various cytokines are closely related to a diverse array of T cell behaviors. Thus, efforts have been made to engineer CAR-T cells with the ability to deliver cytokines *in situ*, or with special cytokine-induced signaling pathways to promote their efficacy. Cytokines can function in paracrine fashion that remodeling the surrounding TME (as introduced in the “Remodeling the immunosuppressive TME” section). They can also directly enhance CAR-T cells in an autocrine manner to boost their activity and expansion. IL-2 is one of the most important cytokines for T cell proliferation and activation. Therefore, the expression of orthogonal human IL-2Rβ on CD19-CAR-T cells allows for controllable expansion upon administration of orthogonal human IL-2, which leads to enhanced antitumor activity against leukemia and lymphoma (86, 87).

Additionally, IL-15 has been shown to promote CD8^+^ T cell activation and proliferation, which can enhance antitumor activity (88). A case report has described the use of CD19-CAR-T cells with membrane-bound IL-15 for B-ALL (following CD19- and CD22-CAR-T cell therapy failure) (89), demonstrating such IL-15-expressing CAR-T cells can be applied to hematological malignancies. These CD19-CAR-T cells with membrane-bound IL-15 are phenotypically similar to stem-cell memory T cells with prolonged proliferating persistence (90). Additionally, CD123- or CLL-1-CAR-T cells that express IL-15 also have a superior capacity of expansion in AML animal models (91, 92). However, this strategy is also associated with tumor necrosis factor α (TNFα)-induced acute toxicity, which requires an adjuvant anti-TNFα treatment (91).

IL-7 is another valuable cytokine, which is required for T cell development, survival, homeostasis and expansion (93). Studies have demonstrated that co-expression of IL-7 and CCL19 prolongs the survival and improves the activity of CD20-CAR-T cells (94). Furthermore, CD19-CAR-T cells expressing IL-7 and CCL19 are currently being explored in a clinical trial for relapsed/refractory B cell lymphoma (NCT04833504). A similar clinical trial for relapsed/refractory DLBCL is to use CD19-CAR-T cells with IL-7 and CCL19 co-expression in combination with an anti-PD1 monoclonal antibody (NCT04381741). Similar strategy has also been proven for T cell adoptive cellular therapy (ACT) (95), which further strengthened the case for IL-7 and CCL19 overexpression in CAR-T cells.

Recently, IL-36γ-secreting CD19-CAR-T cells have been generated to promote CAR-T cell expansion and persistence (in an autocrine manner), resulting in superior anti-tumor activity in mice bearing EL4-CD19^+^ T lymphoblast tumor. Moreover, IL-36γ also activates surrounding antigen presenting cells (APCs) and endogenous T cells, which might even facilitate the formation of an immune memory against antigen-negative tumors (96).

#### 4.1.2 Modulating signaling networks

The provision of a constitutive signal is an option that is being explored to ensure durable CAR-T cell persistence. Toll-like receptor (TLR) adaptor MyD88 and tumor necrosis factor family member CD40 are nonconventional costimulatory molecules for the activation of T cells. Unlike the conventional stimulatory domains like CD28 or 4-1BB commonly used for CAR-T cells, MyD88/CD40 are unable to trigger T cell expansion upon transduction. However, they provide a costimulatory signal to the conventional CAR-T cell activation pathway, which greatly enhances CAR-T cell proliferation and expansion. This effect has been tested in CD123- or CD19-CAR-T cells against hematological malignancies (97, 98). However, MyD88/CD40 co-stimulated CAR-T cell therapy (like IL-15-expressing CAR-T cells) also requires anti-TNFα treatment to prevent severe toxicity (98).

Besides MyD88/CD40, in a murine model of B cell malignancies, miR155 overexpression together with Lysine-specific demethylase 1 (LSD1) knocking down can induce rapid expansion of CD19-CAR-T cells and improve their antitumor function (although the detailed mechanism remains unclear) (99).

#### 4.1.3 Other combinatory therapies

Several combination therapies have been reported to promote *in vivo* CAR-T cell expansion. The well-known immunomodulatory drug, lenalidomide, induces WT1_235-243_/HLA-A*2402-directed CAR-T cell expansion, leading to enhanced anti-tumor activity in mice inoculated with chronic myelogenous leukemia K526 cells (100).

Additionally, CAR-T cell expansion can be induced by vaccination. Vaccination with dendritic cells containing a modified WT1 peptide (WT1_236Y_) has successfully achieved *in vivo* WT1_235-243_/HLA-A*2402-CAR-T cell expansion, which enhanced CAR-T cell efficacy against murine K526 cell tumor model (16). Moreover, cytomegalovirus (CMV)-specific T cells from patients are isolated, enriched and transduced with a CD19-CAR. Therefore, CMV vaccination is able to promote the expansion of such CAR-T cells. These CMV-CD19-CAR-T cells are studied in CD19^+^ tumor bearing murine models, and have potential utility for B cell non-Hodgkin’s lymphoma treatment (101, 102). Similarly, amphiphile-bound ligand of CAR can bind to endogenous albumin upon injection, which traffics to lymph nodes as well as inserts into cell membranes of APCs (103). APCs can present these ligands to CAR-T cells together with endogenous cytokines and costimulation signaling, leading to significant CAR-T cell expansion (including CD19-CAR-T cells). Hence, the amphiphile-bound ligands serve as booster vaccine (104).

### 4.2 Maintenance of CAR-T cell fitness to prolong persistence

CAR-T cell exhaustion and the subsequent reduction of CAR-T cell fitness are among the factors that diminish long-lasting beneficial therapeutic outcomes. T cell fitness and exhaustion are determined by metabolic alterations, transcriptional/epigenetic regulations, treatment strategies, and signaling dynamics. Newly developed approaches that enhance CAR-T cell fitness from the listed aspects are assessed herein.

#### 4.2.1 Regulation of signaling dynamics by CAR design

Proper CAR design plays an essential role in CAR-T cell fitness, as it determines the fate and manner of T cell activation. By limiting the activation level of CAR-T cells, T cell exhaustion is prevented and thereby T cell persistence is maintained (105). While the co-stimulation of CAR-T cells *via* the CD28 pathway can lead to PI3K-AKT activation and rapid T cell exhaustion; 4-1BB-depedent co-stimulation *via* the p38-MAPK pathway can improve mitochondrial biogenesis (106), which enhances CD19- and CD33-CAR-T cell activity and persistence (106–108). However, contrasting results were obtained for CD28-based CD33-CAR-T cells, which are more potent than 4-1BB-based CD33-CAR-T cells against patient-derived xenograft (PDX) models of childhood AML (109). Therefore, the use of CD28-based or 4-1BB-based CARs needs to be evaluated with caution. It is notable that 4-1BB-based CAR-T cells appear to be less hematological toxic than CD28-based CAR-T cells (110), which is perhaps due to their lower activation capacity. Other than 4-1BB, integration of costimulatory domains, ICOS and CD27, can promote CAR-T cell fitness as well (111, 112), although further investigations are required to validate these constructs. Additionally, inclusion of the aforementioned MyD88/CD40 in CAR has also been proposed. However, study has reported that such integration severely affects the stability of CAR (98).

Regions other than the costimulatory domain also play important roles in the regulation of CAR-T cell activation. Increasing TCR affinity above a certain threshold is known to reduce T cell activity (113, 114), which might also be similar for CAR-T cells. Indeed, Ghorashian et al. (115 *)*, generated a CD19-CAR with low affinity to CD19, namely CAT. They demonstrated that this CAT T-cells exhibits improved proliferation and antitumor activity, without a significant increase in toxicity (115). This strategy is proven effective in clinical study as well (NCT02443831), as 12/14 relapsed/refractory pediatric B-ALL patients achieved molecular remission and CAR-T cell persistence was found in 11/14 patients (115). Furthermore, the reduction of hinge domain flexibility also decreases the affinity of CD19-CAR-T cells to CD19, which leads to reduced proinflammatory cytokine secretion without changing their specific cytotoxicity. When being tested in murine model of leukemia, these CAR-T cells with reduced affinity are able to prolong the survival of mice (116). Alternatively, mutation of the immunoreceptor tyrosine-based activation motif (ITAM) region of CD3ζ of CAR also results in lower affinity of CD19-CAR. Therefore, it can modulate CAR-T cell activation and prolong CAR-T cell persistence (117).

Similar efforts to limit CD19-CAR-T cell activation have been made by increasing the hinge and transmembrane region of CAR (such construct is called CD19-BBz (86) CAR) (105). These CAR-T cells are activated at lower levels, proliferate slowly and retain their cytotoxicity. A clinical trial using CD19-BBz (86) CAR-T cells enabled 6/11 patients with B cell lymphoma to achieve complete remission (NCT02842138) (105).

#### 4.2.2 Altering CAR-T cell metabolism to boost fitness

During chronic activation, mitochondrial dysfunction accumulates in CD8^+^ T cells. This is accompanied by a significant increase in reactive oxygen species (ROS), which impairs mitochondrial function and contributes to T cell exhaustion (118). Therefore, methods that optimize metabolism can provide a straightforward mechanism for the improvement of CAR-T cell fitness. The expression of H_2_O_2_ catalase in CAR-T cells can protect CAR-T cells from H_2_O_2_-induced oxidative stress and limit ROS accumulation, leading to maintained fitness (119). Although it was initially validated in solid tumors, similar strategies can be applied in hematological malignancies. It is noteworthy that ROS is not always harmful. Peroxisome proliferator-activated receptor-gamma coactivator (PGC)-1α activation can lead to increased mitochondrial metabolism and ROS accumulation in T cells, improving their antitumor effects (120, 121). Therefore, CD19-CAR-T cells with PGC1α-overexpression are able to improve mitochondrial function and T cells activity (122). The double-edged role of ROS in CAR-T cells probably results from complex influence of ROS on T cells (123). Therefore, further knowledge about the regulation of ROS and T cells is required. Besides ROS metabolism, the inhibition of cholesterol acyltransferase with avasimibe can alter T cell metabolism and increase CD19-CAR-T cells fitness and activity (124).

#### 4.2.3 Modulating transcriptional/epigenetic regulations in CAR-T cells

T cell exhaustion is closely regulated by transcriptional/epigenetic alterations. Recently, a study reported that CD8^+^ CD19-CAR-T cells in ALL patients undergo exhaustion-related DNA methylation (125), thus demethylation therapy might provide means to ensure CAR-T cell fitness. Indeed, Khawanky et al. (126). employed a 5′-Azacitidine (AZA) demethylation therapy in combination with CD123-CAR-T cell therapy against AML. AZA increases the number of Cytotoxic T-lymphocyte-associated protein 4 (CTLA-4)-negative CD123-CAR-T cells, preventing T cell exhaustion, although anti-TNFα treatment may be required to reduce the accompanied toxicity (126). Similarly, DNA methyltransferase 3α (DNMT3A) deletion in CD19-CAR-T cells also prevents T cell exhaustion and improves CAR-T cell efficacy (127).

#### 4.2.4 Other treatment strategies to preserve CAR-T cell fitness

Anti-cancer drugs can impact CAR-T cell fitness too, although the precise mechanisms remain unclear. An *in vitro* study demonstrated that JQ1, an epigenetic inhibitor, reverses the exhaustion of CAR-T cells obtained from nonresponding chronic lymphocytic leukemia patients (128). Ibrutinib, a bruton tyrosine kinase inhibitor for mantle cell lymphoma and chronic lymphocytic leukemia, was also shown to increase CD19-CAR-T cell fitness against leukemia (129, 130).

The possibility of rejuvenating exhausted CAR-T cells has also been explored. Luo et al. (131) constructed a fluorescein-targeted-CAR-T cells with both antigen recognition and drug internalization ability. These CAR-T cells were directed toward the folate receptor^+^ cancer cells *via* a fluorescein-folate bispecific adaptor. Upon CAR-T cell exhaustion, TLR7-1A linked fluorescein is administered and then internalized by fluorescein-CAR-T cells, leading to their reactivation. This type of strategy could also be applied to hematological malignancies *via* the use of a bispecific adaptor targeting CD19 or CD22.

### 4.3 Conferring stemness on CAR-T cells

Clinical studies have indicated that CAR-T cells with a less-differentiated phenotype (such as memory stem cell-like T cells, T_SCM_-like) can exhibit longer proliferation and superior antitumor function (132–134). Therefore, generating CAR-T cells in a less differentiated state can guarantee their persistence and enormous achievements have been made in recent years. Although these protocols vary in different platforms, the key step is to inhibit terminal differentiation of CAR-T cells. While IL-21 plays a key role in the maintenance of memory T cells (including T_SCM_ and central memory T cells, T_CM_) by inducing naïve T cells into an early differentiation phenotype (135), IL-15 prevents T cell differentiation by inhibiting mTORC1 (85). Therefore IL-21 or IL-15 were commonly administrated during manufacturing. Indeed, several studies reported that the addition of IL-21 or IL-15 results in increased proportion of CAR-T_SCM_ and T_CM_ (including WT1- and CD19-CAR-T cells) associated with enhanced persistence and anti-tumor effects (85, 136–140). Furthermore, induced secretion of IL-21 by CD19-CAR-T cells can even maintain CAR-T cells in early memory phenotype and enhance anti-tumor activity *in vivo*, which was already demonstrated in mice transplanted with Ramos B cells (139).

Besides cytokine administration, other methods could be applied to move CAR transduction toward a less differentiated phenotype. Upon costimulatory molecule CD81-mediated *ex vivo* T cell activation during manufacturing, CD19-CAR-T cells are enriched with naïve T cell-derived. These T cells are less differentiated with increased CAR expression and antitumor activity (141). Moreover, CAR-T cell stemness can also be maintained by modulating signaling pathways. PI3K or AKT inhibition can preserve CAR-T cell persistence by preventing T cell differentiation. Therefore, CAR-T cells are maintained in a less differentiated state, as demonstrated in CD33- (142), CD5- (143), CD19- (144, 145) and Minor histocompatibility antigen (MiHA)- (146) CAR-T cells against various hematological malignancies. Besides the PI3K-AKT pathways, the modulation of other pathways could maintain CAR-T cells memory stem cell-like features and even revive exhausted CAR-T cells, including Notch activation (147) and tyrosine kinase inhibition (148, 149) for CD19-CAR-T cells against humanized murine leukemia model. Recently, the role of MEK inhibition in CD8^+^ T_SCM_ formation has been discovered (150), making it another strategy for CAR-T_SCM_ generation.

### 4.4 Novel factors affecting CAR-T cell persistence

Besides exhaustion, CAR-T cells also undergo activation-induced cell death (AICD) upon repeated activation, which is a manner of cell apoptosis through Fas-dependent pathways (151). Methods of Fas blockade or limiting CAR activation were introduced in previous sections (“Targeting immunosuppressive effects (other than immune checkpoints)” and “Regulation of signaling dynamics by CAR design”). ACID can also be prevented by Bcl-2 activation. CD20-CAR-T cells with Bcl-2 overexpression exhibit enhanced viability and antitumor activity in mouse xenograft lymphoma model (152).

As discussed above, many strategies that enhance CAR-T cell activity and expansion also increase the risk of adverse effect, some even need adjuvant treatment. However, as lessons learnt from HSCT, the onset of a mild graft-versus-host disease (GVHD, the major complication) may be associated with a lower risk of disease relapse after HSCT (153). Indeed, enhanced function of T cells probably leads to improved immune response toward both cancer cells and health cells. Therefore, adverse effects might not be solely a bad thing and researchers should treat them accordingly. Nevertheless, some adverse effects of CAR-T cells are life-threatening and require special attentions. These will be covered in the next sections.

## 5 Mitigating toxic adverse effects associated with CAR-T cell therapy

Despite the superior therapeutic success and efficacy that have been achieved by CAR-T cell treatment, several concerning adverse effects require special attention, including: cytokine release syndrome (CRS)/neurotoxicity, on-target-off-tumor effects and off-target effects. These adverse effects greatly limit patient prognosis and restrict the outcomes associated with CAR-T cell therapy. Extensive efforts have been made to minimize these adverse effects. In the following sub-sections, we will focus on the strategies that directly modify CAR-T cells to minimize unintended toxicity and related preclinical studies.

### 5.1 The direct management of CRS/neurotoxicity during CAR-T cell therapy

Upon CAR-T cell activation, massive cytokines are produced by CAR-T cells and bystander cells. High concentrations of circulating pro-inflammatory cytokines can lead to the hyperactivation of the cytokine signaling network, and potentially lead to cytokine toxicity or CRS (154). It has been suggested that the presence and severity of CRS may be related to the treatment outcomes (155), but this notion remains controversial. Nevertheless, CRS is a life-threatening symptom and requires urgent therapeutic interventions (156). Immune effector cell-associated neurotoxicity syndrome (ICANS) is the second most frequent adverse effect associated with CAR-T cell therapy. Its underlying mechanisms appear to be similar to that of CRS, i.e., Both of them are due to the action of CAR-T cells and bystander cells (157). Therefore, numerous improved CAR-T cell designs and treatment strategies have been proposed to mitigate CRS/neurotoxicity by avoiding excessive cytokine release. IL-6 and IL-1 are closely related to CRS (158–160). Therefore, CD19- or BCMA-CAR-T cells with the capacity of anti-IL-6 scFv and IL-1 receptor antagonist (IL-1RA) secretion can help self-neutralize IL-6 and IL-1β. CRS and neurotoxicity are minimized in patients treated with these anti-IL-6 scFv and IL-1RA-secreting CAR-T cells (ChiCTR2000032124; ChiCTR2000031868) (161). Besides IL-6 and IL-1, cytokine-profiling assay identified granulocyte-macrophage colony-stimulating factor (GMCSF) as a key CRS-promoting protein released from CAR-T cells (162). Therefore, the reduction of GMCSF from CAR-T cells *via* antibody-mediated neutralization or GMCSF knockout significantly decreases the secretion of key CRS-associated cytokines from CAR-T cells without affecting their antitumor function, as shown in CD19-CAR-T cells in murine leukemia model (163) and CD22-CAR-T cells (162).

IFNγ inhibition is considered to be a possible salvage option for refractory CRS when IL-6 inhibition and glucocorticoids treatment fail. Whilst IFNγ production is an indicator of CAR-T cell activity, IFNγ blockade or deletion do not significantly impair the antitumor effect of CD19-CAR-T cells in murine models. Moreover, IFNγ removal results in attenuated cytokine toxicities due to reduced bystander macrophage activation. It also reduces the immune checkpoints expression on CAR-T cells and ensures their persistence (164). Moreover, it should be noted that IFNγ blockade is only applicable in CAR-T cells against hematological malignancies, as IFNγ receptor pathway is required for CAR-T cell killing activity in solid tumors (165).

### 5.2 Eliminating on-target off-tumor effects of CAR-T cells

For most forms of cancers, it is rare to find tumor-specific antigens that are exclusively expressed in cancer cells and shared by most cancer patients. Therefore, on-target off-tumor toxicity is a major concern of CAR-T cell therapy as the target antigens are also expressed on normal tissues. The development of single cell sequencing might improve the target antigen analysis, and provide valuable guidance on on-target, off-tumor toxicity evaluation (166). Nevertheless, several strategies have been proposed to attenuate or mitigate on-target off-tumor effect of CAR-T cells, including refined T cell regulation and the administration of preventive measures.

#### 5.2.1 Gating strategies for CAR-T cells

To avoid the cross-reactivity on normal tissues, a variety of gating strategies of AND, OR and NOT have been implemented for CAR-T cells for a long time. In recent years, several studies have proposed further improvements to this gated system for a more refined regulation of CAR-T cell activity.

AND-gated CAR-T cells have two (or more) CAR-like receptors and are only activated upon two (or more) antigens binding. Further increased modulation of this AND-gated CAR-T cell system can be achieved by the incorporation of a synthetic Notch receptor. The synthetic Notch receptor recognizes the first antigen and triggers the expression of a second CAR that recognizes another antigen (167). Roybal et al. (167) introduced the use of CAR-T cells with a CD19-synNotch receptor and an inducible α-mesothelin CAR gene *in vitro*. These CAR-T cells are not activated in the presence of mesothelin alone, they need to encounter CD19 first to express the α-mesothelin CAR. The author further demonstrated that synNotch receptors are able to induce tumor-localized CAR expression *in vivo*, by using CAR-T cells with GFP synNotch receptor and inducible CD19-CAR gene against GFP^+^ CD19^+^ tumor cells (167).

NOT-gated CAR-T cells, or inhibitory CAR (iCAR) T-cells are CAR-T cells with an activating receptor and an inhibitory receptor. The inclusion of an inhibitory receptor that targets antigens expressed on normal tissues minimizes the potential for cross reactivity. For example, CAR-T cells with CD19-CAR and HLA-A*02-iCAR have been constructed (168). Such CAR-T cells exploit the loss of heterozygosity (LOH) commonly observed in tumors. The presence of HLA-A*02-iCAR allows CAR-T cells to selectively eliminate hemizygous lymphoma cells which lost HLA-A*02 *via* LOH among heterozygous normal cells. By switching different types of HLA-iCAR, this approach can be extended to patients beyond HLA-A*02 heterozygotes.

And-, NOT- and OR-gated (the OR-gated strategy, i.e., the bispecific CAR) can be combined to produce more complex/refined regulation networks. Williams et al. (169) developed a system with multiple input circuits, which enables the production of CAR-T cells with complex activation mode like “A and B and C”, “A and B not C” or “[A or B] and C”. Moreover, multiple synNotch receptors have also been included in this system. Several combinations have been investigated, including an HER2-NOT-gate with an AND-gate of CD19 and GFP against K562 cells. However, most of the gated designs in this study were only tested *in vitro* and *in vivo* for K562 cells engineered to express different combinations of the antigens. Whether such multiple input circuits system can be applied to unmodified or spontaneous tumor models requires further investigation.

#### 5.2.2 Preventive measures for on-target off-tumor toxicity

Preventive measures provide an alternative approach that can be applied to relieve damage incurred by on-target off-tumor toxicity. For example, whilst CD7-CAR-T cells are able to target T-ALL, normal CD7^+^ T cells and NK cells are eliminated as well, leading to immunodeficiency. To protect normal cells, Kim et al. genetically deleted CD7 in hematopoietic stem cells (HSCs) which were then engrafted into mice prior to the CD7-CAR-T cell treatment. These CD7-KO HSCs generated functional CD7^-^ T cells and NK cells, although they are different from CD7^+^ T cells/NK cells. As the results, the mice are protected from CD7-CAR-T cell-induced immunodeficiency (170). Similarly, infusion of CD33-KO HSCs is able to restore immunity upon CD33-CAR-T cell treatment against AML (171, 172). Although the infusion of these antigen-removed HSCs provides an ideal strategy to maintain hematopoiesis during CAR-T cell therapy, concerns about the safety of genetic modification and phenotype of the antigen-removed HSCs exist. Moreover, in case of antigen-negative relapse and a second type of CAR-T cells is need, these antigen-removed HSCs become useless. It will only be applicable to clinics after all related techniques are mature.

### 5.3 CAR-T cells with On-switch

The development of an On-switch for CAR-T cells enables the tight regulation of CAR-T cell activity and toxicity. Recently, On-switch system which takes the advantage of the tetracycline-on (Tet-On) system (173) has been developed. In the presence of doxycycline (Dox), reverse Tet transactivator (rtTA) fusion protein drives the expression of protein of interest. This Tet-On system has been applied to CD19-CAR-T cells against leukemia cells (174), and CD38-CAR-T cells against MM cells (175). In these studies, peak CAR expression is observed within 24 to 48 hours of Dox administration; and upon Dox withdrawal, CAR expression decay occurs within 24 to 48 hours. As Dox is generally well tolerated and allergic reactions are uncommon (176), this system may have a wide application.

Hypoxia is closely related to gene regulation and is a common feature shared by various TME. Therefore, a hypoxia-inducible transcription amplification system (HiTA-system) has been developed to control the expression of CAR in T cells (HiTA-CAR-T) (177). He et al. (177) have demonstrated that Her2-directed HiTA-CAR expression and HiTA-CAR-T activity are tightly restricted to hypoxic environments, with nearly no CAR expression in normoxic tissues in a mouse xenograft liver cancer model (177). As bone marrow niches are hypoxic, this technique has great potential for the treatment of hematological malignancies as well.

The light-switchable CAR (LiCAR) T-cells is a light-controlled system of On-switch. The intracellular domains of CAR are split into half, each half with a photoresponsive module. The two halves of CAR can only reassemble in the presence of near-infrared (NIR)-to-blue light, thereby allowing the transduction of T cell activation signals. This spatial-temporal regulation system can achieve attenuated toxicity and has been tested for CD19-CAR-T cells against lymphoma both *in vitro* and *in vivo* (178). Moreover, Kobayashi et al. (179) introduced a light-controlled binary switch system for CAR-T cell activation. They used the aforementioned fluorescein-directed CAR-T cells and an adaptor in which fluorescein is conjugated to small molecules targeting CD38. Therefore, the CAR-T cell activity is directed to CD38^+^ Ramos B cells *in vitro*. Such adaptors are initially trapped in an ultraviolet (UV)-light-sensitive cage. They are released upon UV exposure to induce CAR-T cell function *in vitro* (179).

Besides light, light-induced heat serves as another means of spatial-temporal CAR expression modulation, which is developed by Miller et al. (180). In this system, CD19-CAR expression is controlled by synthetic gene switches. When temperature reaches 40-42°C by photothermal heating, CAR expression is induced. In murine models with B cell driven cancer (i.e., Raji cells), plasmonic gold nanorods (AuNRs) were injected intravenously to convert NIR light into heat, together with heat-controlled CD19-CAR-T cells. When heating with NIR laser light directly to induce local CAR expressions, CAR was expressed in T cells and the tumors were irradiated with enhanced safety and efficacy (180). The major concern of these light/heat-based systems is that whether light/heat can penetrate into body. It is unlikely for a UV-based system (179) to reach site of hematological malignancies. By contrary, deep-tissue-penetrable NIR light is able to directly target tumor sites for precise control of CAR-T cells, as demonstrated in murine models of lymphoma (178, 180). However, further investigations are required to explore whether NIR light-controlled CAR-T cells are applicable in large and complex human body.

### 5.4 CAR-T cell control using an Off-switch

#### 5.4.1 CAR-T cell elimination

Besides switches that activate CAR-T cells, CAR-T cell activity can be switched off by the incorporation of a suicide genes. By overexpressing an inducible caspase9 (iCasp9 by AP1903 induction), majority of CD20- or CD19-CAR-T cells are eliminated within 72 hours upon AP1903 administration (181, 182). Other studies have further demonstrated that it improves CAR-T cell therapy safety through the use of iCasp9 in CD33-CAR-T cell against AML (183), interleukin-1 receptor accessory protein (IL-1RAP)-CAR-T cell against chronic myeloid leukemia (184) and CD19-CAR-T cell against various hematological malignancies (98).

The binding of monoclonal antibodies to cells induces antibody-dependent cellular cytotoxicity (ADCC) and complement-dependent cytotoxicity, leading to cell elimination. This phenomenon has been exploited for CAR-T cell elimination and the overexpressed CD20 or CD34 in lymphocytes are good candidates for elimination targets (185–187). For example, Sommer et al. (188) developed a rituximab-base (targeting CD20) off-switch system to eliminate FLT3-CAR-T cells in AML models. The application of this strategy enables rapid elimination of CAR-T cells to enhance bone marrow recovery without impairing antitumor activity (188). However, as CAR-T cells for hematological malignancies are likely to impair immunity, whether patients can survive the side effects of another lymphocyte-targeting treatment requires careful evaluation.

#### 5.4.2 Off-switch by CAR expression regulation

The small molecule–assisted shutoff (SMASh) system was firstly developed by Chung et al. (189), which offers another strategy to eliminate CAR-T cells. In this system, proteins of interest are fused to a SMAsh tag (consisting of an HCV protease and a degron) at the C-terminal *via* HCV NS3 protease recognition site. After protein folding, the internal protease cleaves at the fusion site to release the protein of interest, while the SMAsh tag is degraded due to degron activity. HCV protease inhibitors can block tag removal and the degron then leads to rapid degradation of the protein-tag fusion (189). This SMAsh strategy was implemented into CAR construct (SWIFF-CAR) by Juillerat et al. (190). In the absence of asunaprevir (a protease inhibitor), CAR is normally expressed on the cell surface *in vitro*. Upon administration of asunaprevir to the medium, the protease activity is inhibited within 48 hours, leading to rapid degradation of the newly synthesized CAR (190). Cao et al. (191) further applied this SMAsh technique *in vivo*: CD19-CAR-T cells with the SMAsh tag can efficiently kill human CD19^+^ tumor cells in mice. They also demonstrated that asunaprevir administration/withdrawal can control repeated surface CAR expression in a dose-dependent manner (191). Such repeated control of CAR expression might be helpful to avoid chronic antigen stimulation and prevent CAR-T cell exhaustion.

## 6 Novel approaches of CAR-T cell therapy

### 6.1 CAR-T cells as activator of prodrug

As discussed above, the current limitations of CAR-T cells include antigen-negative relapse, immunosuppressive TME, poor persistence and severe toxicities. To address these limitations, Gardner et al. (192) developed a novel class of CAR-T cells, namely synthetic enzyme-armed killer (SEAKER) cells. By taking advantages of the high specificity of CAR-T cells, engineered SEAKER cells which express prodrug-activating enzymes can activate systemically administered small molecule prodrugs specifically at tumor site. In murine lymphoma and leukemia models, CD19-directed SEAKER cells exhibited enhanced anti-tumor activity which is due to both direct CAR-T cell intrinsic function and activated prodrugs. Moreover, activation of prodrug allows CD19-directed SEAKER cells to kill CD19-negative cancer cells as well, making this strategy possible to eradicate heterogeneous tumor and prevent antigen-negative relapse. In addition, prodrug-activating capability is maintained in exhausted CD19-SNEAKER cells after multiple courses of prodrug treatment, indicating the superior persistence of this therapy. Finally, as prodrugs activation is independent of the immune activity of SEAKER cells, it is not affected by the immunosuppressive TME and the dose of infused SEAKER cell can be reduced to minimize immune toxicity (192). Therefore, SEAKER cell is an excellent approach to address multiple major limitations of CAR-T cells in hematological malignancies.

### 6.2 Tackling fratricide of CAR-T cells

Currently, majority of CAR-T cell therapies for hematological malignancies are against B cell driven cancers. A major difficulty of the development of CAR-T cells against T cell driven cancers is that the attractive antigens (such as CD7) are expressed on normal T cell surfaces as well, leading to potential fratricide of CAR-T cells (i.e., CAR-T cells attack each other due to the presence of CD7). Such fratricide can be avoided through genetic modification of CD7 in CAR-T cells (193, 194). However, Freiwan et al. (195), developed a modification-free approach by isolating the naturally occurring CD7-negative T cells to generate CD7-negative CD7-CAR-T cells. These CD7-negative CD7-CAR-T cells closely resemble CD4^+^ memory T cells and exhibit promising persistence. Moreover, CD7-negative CD19-CAR-T cells also show enhanced anti-tumor activity compared to normal CD19-CAR-T cells. Therefore, CD7-negative T cells may be an ideal source of CAR-T cells for T cell driven cancers and other hematological malignancies.

Besides CD7-negative CD7-CAR-T cells, naturally selected CD7-CAR (NS7CAR)-T cells may be another promising modification-free therapy against T cell driven cancers. These CD7-negative NS7CAR-T cells were generated from fratricidal “natural selection” after the transduction of CD7-CAR constructs into bulk T cells. When compared to CD7-negative CD7-CAR-T cells, NS7CAR-T cells exhibited similar anti-tumor properties and have already passed phase 1 clinical trial recruiting patients with T-ALL or T-cell lymphoblastic lymphoma (NCT04572308) (196). However, extensive immune response occurs during “natural selection”. Whether this leads to pre-mature exhaustion of NS7CAR-T cells requires further study.

## 7 Conclusion

**Figure 3:**
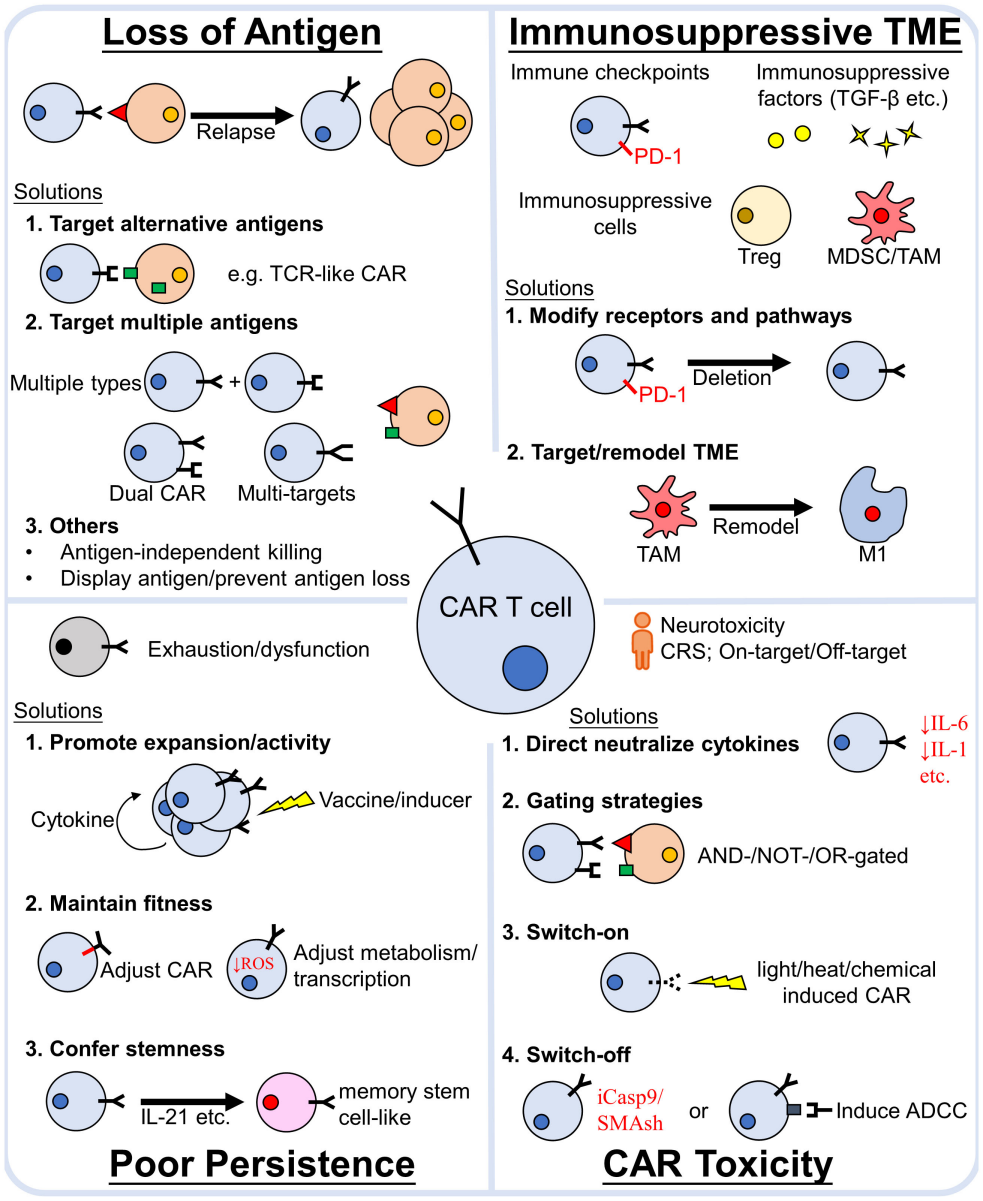
Major causes of CAR-T cell treatment failure and strategies to avoid them. The main reasons for CAR-T cell treatment failure are (1) loss of antigen relapse, (2) the immunosuppressive TME, (3) poor CAR-T cell poor persistence *in vivo* and (4) severe toxicity. Numerous efforts have been made to propose strategies to overcome these obstacles. The efficacy of CAR-T cell therapy can be enhanced by addressing these factors. TCR, T-cell receptor; TME, tumor microenvironment; PD-1, Programmed death-1; TGF-β, transforming growth factor -β; MDSC, myeloid-derived suppressor cells; TAM, tumor associated macrophages; Treg, regulatory T cells; CRS, cytokine release syndrome; iCasp9, inducible caspase9; SMAsh, Small molecule–assisted shutoff; ADCC, antibody-dependent cellular cytotoxicity.

CAR-T cell therapy has revolutionized both the field of immunotherapy as well as the treatment of hematological malignancies. Although limitations still exist, great endeavor has been done to address them. Continuous development and improvement of antigen discovery, together with strategies to avoid antigen loss, impart us an arsenal to tackle tumor heterogeneity and antigen-negative relapse. Barriers of the immunosuppressive TME can be overcame *via* circumventing, targeting or remodeling TME. In addition, CAR-T cell persistence inside patient’s body is also crucial for a durable response of the treatment and it can be prolonged by proper CAR construct design, as well as ensuring CAR-T cell expansion, fitness and stemness. Moreover, strategies to enhance, refine and control CAR-T cell activity through various switch or gated systems are being established to mitigate the adverse effects during CAR-T cell therapy (**Figure 3**).

CAR-T cell therapy is a booming field that incorporates both fundamental and clinical researches. Its great success in certain hematological malignancies has completely reshaped the approach to cancer and cancer therapies. The sophisticated immune evasion strategies of cancer cells and the complex immune microenvironment still limit CAR-T cell activity, but we are on the right track to unveil them. Fortunately, numerous solutions are already on the horizon. We envision that there will be a giant leap for CAR-T cell therapy once these recently developed preclinical improvements are translated into clinics. Besides the aspects related to the direct modifications on CAR-T cells discussed in this review, other factors that need to be further addressed for CAR-T cell therapy include: improving manufacturing practices with a shorter processing time, allogeneic or off-the-shelf CAR-T cells, improving CAR-T cell infiltration for solid tumors, pre-conditioning strategies for patients and the use of other immune cells for CAR transduction (e.g. NK cells, macrophages etc.). With advances in the understanding of cancer biology, immune systems and bioengineering/biomaterials, ways have been paved for the rapid developments of CAR-based therapies from different disciplines. We hope this review may provide new viewpoints for multidisciplinary approaches and the obstacles of effective CAR-T cell therapy will be overcome in the near future.

## Author contributions

Conceptualization, JWH and JH; Investigation, JWH; Writing - Original Draft, JWH; Writing -Review and Editing, JWH, XH, and JH; Supervision, JH. All authors contributed to the article and approved the submitted version.

## Funding

This work was financially supported by National Natural Science Foundation of China (NSFC 81500173).

## Acknowledgments

We acknowledge Dr. Yangbao Miao for his ongoing advices and Dr. Yiguo Hu for language editing. We apologize to colleagues whose work we could not cite due to space limitations.

## Conflict of interest

The authors declare that the research was conducted in the absence of any commercial or financial relationships that could be construed as a potential conflict of interest.

## Publisher’s note

All claims expressed in this article are solely those of the authors and do not necessarily represent those of their affiliated organizations, or those of the publisher, the editors and the reviewers. Any product that may be evaluated in this article, or claim that may be made by its manufacturer, is not guaranteed or endorsed by the publisher.

## Glossary

**Table:**

4-1BB	CD137, Tumor necrosis factor receptor superfamily 9
ACT	adoptive cell therapy
ADCC	antibody-dependent cellular cytotoxicity
AICD	activation-induced cell death
ALL	acute lymphoblastic leukemia
AML	acute myeloid leukemia
APC	antigen presenting cell
ATRA	all-trans retinoic acid
AuNR	plasmonic gold nanorod
AZA	5′-Azacitidine
BAFF	B-cell activating factor
BCMA	B cell maturation antigen
CAF	cancer-associated fibroblast
CAR	chimeric antigen receptor
CLL-1	C-type lectin-like molecule-1
CMV	cytomegalovirus
CRS	cytokine release syndrome
CTLA-4	Cytotoxic T-lymphocyte-associated protein 4
D2HG	D-2-hydroxyglutarate
D2HGDH	D2HG dehydrogenase
DLBCL	diffuse large B-cell lymphoma
DNMT3A	DNA methyltransferase 3α
Dox	doxycycline
FA-TLR7-1A	folate-targeted Toll-like receptor 7 agonist
FDA	Food and Drug Administration
GMCSF	granulocyte-macrophage colony-stimulating factor
GVHD	graft-versus-host disease
GVL	graft-versus-leukemia
GVT	graft-versus-tumor
HiTA	hypoxia-inducible transcription amplification
HLA	human leukocyte antigens
HMHA1	Minor histocompatibility antigen 1
HSA	IL-12-loaded human serum albumin
HSC	hematopoietic stem cell
HSCT	hematopoietic stem cell transplantation
ICANS	immune effector cell-associated neurotoxicity syndrome
iCAR	inhibitory CAR
iCasp9	inducible caspase9
ICV	intracerebroventricular
IFNγ	interferon γ
IL-1RA	IL-1 receptor antagonist
IL-1RAP	interleukin-1 receptor accessory protein
INS-CAR T	IL-12 nanostimulant-engineered CAR-T cell
ITAM	immunoreceptor tyrosine-based activation motif
IV	intravenous
LiCAR	light-switchable
LOH	loss of heterozygosity
LSD1	Lysine-specific demethylase 1
MDSC	myeloid-derived suppressor cells
MiHA	Minor histocompatibility antigen
MM	multiple myeloma
NIR	near-infrared
NK	natural killer
NS7CAR	naturally selected CD7-CAR
PD-1	Programmed death-1
PGC	Peroxisome proliferator-activated receptor-gamma coactivator
PI3K	Phosphoinositide 3-kinase
PD-L	Programmed death-1 ligand
RASA2	RAS P21 Protein Activator 2
rtTA	reverse Tet transactivator
scFv	single chain variable fragments
SEAKER	synthetic enzyme-armed killer
SMASh	small molecule–assisted shutoff
TACI	Transmembrane activator and CAML interactor
TAM	tumor associated macrophages
T_CM_	central memory T cell
TCR	T-cell receptor
Tet-On	tetracycline-on
TGF-β	transforming growth factor beta
TIGIT	T cell immunoreceptor with Ig and ITIM domains
TIM-3	T-cell immunoglobulin and mucin-domain containing-3
TLR	toll-like receptor
TME	tumor microenvironment
TNFα	tumor necrosis factor α
T_SCM_	memory stem cell-like T cell
UV	ultraviolet
WT1	Wilms tumor 1
